# A Winter Distribution Model for Bicknell’s Thrush (*Catharus bicknelli*), a Conservation Tool for a Threatened Migratory Songbird

**DOI:** 10.1371/journal.pone.0053986

**Published:** 2013-01-09

**Authors:** Kent P. McFarland, Christopher C. Rimmer, James E. Goetz, Yves Aubry, Joseph M. Wunderle, Anne Sutton, Jason M. Townsend, Alejandro Llanes Sosa, Arturo Kirkconnell

**Affiliations:** 1 Vermont Center for Ecostudies, Norwich, Vermont, United States of America; 2 Natural Resources Department, Cornell University, Ithaca, New York, United States of America; 3 Environment Canada, Canadian Wildlife Service, Ste-Foy, Canada; 4 International Institute of Tropical Forestry, USDA Forest Service, Sabana Field Research Station, Luquillo, Puerto Rico, United States of America; 5 Marshall’s Pen, Mandeville, Jamaica; 6 University of California Davis, Department of Wildlife, Fish and Conservation Biology, Davis, California, United States of America; 7 Instituo de Ecología y Sistematica, La Habana, Cuba; 8 Museo Nacional de Historia Natural de Cuba, La Habana, Cuba; Monash University, Australia

## Abstract

Conservation planning and implementation require identifying pertinent habitats and locations where protection and management may improve viability of targeted species. The winter range of Bicknell’s Thrush (*Catharus bicknelli*), a threatened Nearctic-Neotropical migratory songbird, is restricted to the Greater Antilles. We analyzed winter records from the mid-1970s to 2009 to quantitatively evaluate winter distribution and habitat selection. Additionally, we conducted targeted surveys in Jamaica (*n* = 433), Cuba (*n* = 363), Dominican Republic (*n* = 1,000), Haiti (*n* = 131) and Puerto Rico (*n* = 242) yielding 179 sites with thrush presence. We modeled Bicknell’s Thrush winter habitat selection and distribution in the Greater Antilles in Maxent version 3.3.1. using environmental predictors represented in 30 arc second study area rasters. These included nine landform, land cover and climatic variables that were thought *a priori* to have potentially high predictive power. We used the average training gain from ten model runs to select the best subset of predictors. Total winter precipitation, aspect and land cover, particularly broadleaf forests, emerged as important variables. A five-variable model that contained land cover, winter precipitation, aspect, slope, and elevation was the most parsimonious and not significantly different than the models with more variables. We used the best fitting model to depict potential winter habitat. Using the 10 percentile threshold (>0.25), we estimated winter habitat to cover 33,170 km^2^, nearly 10% of the study area. The Dominican Republic contained half of all potential habitat (51%), followed by Cuba (15.1%), Jamaica (13.5%), Haiti (10.6%), and Puerto Rico (9.9%). Nearly one-third of the range was found to be in protected areas. By providing the first detailed predictive map of Bicknell’s Thrush winter distribution, our study provides a useful tool to prioritize and direct conservation planning for this and other wet, broadleaf forest specialists in the Greater Antilles.

## Introduction

Bicknell’s Thrush (*Catharus bicknelli*) is among North America’s most rare, range-restricted breeding passerines. Considered one of the Nearctic-Neotropical migrants at greatest risk of extinction and thus of highest continental conservation concern [1]–[4], Bicknell’s Thrush is classified as globally “vulnerable” by the International Union for the Conservation of Nature (IUCN) and Threatened in Canada [5], [6].

Bicknell’s Thrush is a habitat specialist that occupies a naturally fragmented breeding range from the Catskill Mountains of New York northeastward to the Gulf of St. Lawrence and Cape Breton Island, Nova Scotia [2], [7]. In New York, northern New England and the nearby Estrie region of Québec, Bicknell’s Thrush inhabits montane forests dominated by balsam fir (*Abies balsamea*), with lesser amounts of spruce (*Picea rubens* and *P. mariana*), white birch (*Betula papyrifera* var. *cordifolia*), and mountain ash (*Sorbus americana* and *S. decora*) [2], [7], [8].

At both ends of its migratory range, Bicknell’s Thrush occupies a limited, highly fragmented distribution and faces multiple habitat threats that may impact populations [2], [7], [9]. These include climate change [10], acid ion deposition [11]–[13], mercury contamination [14], mountaintop development [2], [15], [16], forestry operations [17], [18], and loss and degradation of winter habitats [2], [19].

Breeding population trend data for Bicknell’s Thrush are sparse but generally indicate declines, especially in core and northern parts of the range. Breeding Bird Survey data on 16 routes in Canada from 1968–2008 showed a significant decline of 9% per year [6]. Trail-based point count surveys in the White Mountains of New Hampshire from 1993–2003 revealed a 7% annual decline (*P*<0.1) [20]. The High Elevation Landbird Program documented significant (P<0.05) annual declines of 17% in New Brunswick and 15% in Nova Scotia from 2002–2009 [21]. The species’ overall pattern of rangewide declines suggests that its populations are strongly limited at one or more stages of its annual cycle. Recent evidence indicates that a complex interplay of ecological and demographic factors on the wintering grounds, exacerbated by forest loss and degradation, may be a primary limiting factor for populations of Bicknell’s Thrush [22], [23].

Despite considerable attention currently focused on Bicknell’s Thrush, surprisingly few empirical data exist by which to evaluate the species’ conservation status on its Caribbean wintering grounds. Until recently, the winter range was imprecisely known, due to a paucity of records. Wetmore and Swales [24] provided the first documentation of the species’ limited Greater Antillean distribution, when they examined specimens previously assigned to Gray-cheeked Thrush subspecies (C. *minimus minimus* and *C. m. alicea*). Careful review of these and other specimen records by Wallace [25] revealed only a single specimen from Haiti and six from the Dominican Republic. He speculated that “perhaps they also inhabit suitable locations on other West Indian islands, such as the mountains of eastern Cuba, Jamaica and Puerto Rico, but no specimens have been taken to substantiate this assumption.” Wallace’s conjecture was subsequently verified by records from Cuba [26], [27] and mist-net captures in Puerto Rico (W. Arendt, unpubl. data).

We initiated targeted surveys to clarify the winter distribution and habitat use of Bicknell’s Thrush on Hispaniola in the mid-1990s and expanded these to other islands in the Greater Antilles during the following decade. Rimmer et al. [2] summarized extant rangewide information but acknowledged that significant gaps remain. In this study we analyze an extensive dataset of Bicknell’s Thrush winter records compiled from the mid-1970s to 2009, and we present the first quantitative evaluation of the species’ winter distribution and habitat selection. Our specific objectives were to (1) conduct standardized field surveys to document the presence and presumed absence of Bicknell’s Thrush throughout the Greater Antilles, (2) relate the species’ occurrence to a suite of habitat, geographic, topographic, and climatic variables, (3) compare current distribution data with historic information, (4) create a predictive model of Bicknell’s Thrush distribution and habitat selection, and (5) use the model’s habitat projections to refine assessments of the species’ winter conservation status and develop conservation recommendations.

## Materials and Methods

### Data and Study Area

We compiled all known historic (pre-1994) and recent (post-1994) winter (November to April) sightings, banding records, and specimen records of Bicknell’s Thrush from the literature and from personal correspondence with ornithologists and other field researchers (*n* = 15 records). We treated reports of one or more thrushes detected at a particular site as a single record. Because we found no reliable evidence for Bicknell’s Thrush winter records outside of the Greater Antilles, we restricted our study area to the islands of Cuba, Hispaniola, Jamaica, and Puerto Rico. We included in our analyses only those records that could be geographically assigned with confidence to a ∼1 km^2^ pixel on our study area grid.

From 1994–2009, we conducted targeted presence-presumed absence surveys for Bicknell’s Thrush at 2,169 points, each separated by ≥100 m. Surveys were conducted in Jamaica (1996–1998; *n* = 433 points), Cuba (1998–2005, *n* = 363), the Dominican Republic (1994–2009; *n* = 1000), Haiti (2004–2007, 2009; *n* = 131) and Puerto Rico (1996; *n* = 242). Because of the large extent of our study area, the relative rarity of Bicknell’s Thrush, and the fact that most previously published winter records for the species were from moist broadleaf or dense pine-broadleaf mixed forests, we restricted the majority of surveys to these forest types. However, we also surveyed other wooded habitats, including dry and semi-mesic broadleaf forests, pine forests, mangroves, and shade coffee and cacao plantations. Surveys were conducted from dawn until mid-morning and near dusk, when thrushes were most active and often spontaneously vocalized. At each survey point, observers used conspecific playbacks of calls and songs to elicit responses from Bicknell’s Thrushes.

More detailed habitat metrics were recorded from 1994–1998 for a subset of survey points (*n = *874) in the Dominican Republic, although habitat data were not collected for all variables at all points. Ocular estimates of canopy height (m), understory and canopy density (% cover), forest type (broadleaf, pine, mixed, scrub-shrub), seral stage (old growth, old growth-secondary, secondary), and moisture gradient (dry to wet) were determined at most points.

### Predictive Modeling

We modeled Bicknell’s Thrush winter habitat selection and distribution in the Greater Antilles using Maximum Entropy Modeling of Species Geographic Distributions version 3.3.1 (Maxent; http://www.cs.princeton.edu/~schapire/maxent). Phillips et al. [28] and Philips and Dudik [29] provide a complete formulaic and software description of Maxent as used in this study. Maxent is based on a machine learning response that starts with known locations and compares environmental correlates at those sites to these same correlates at 10,000 random points throughout a given study area. Maxent estimates the most uniform distribution, the maximum entropy, of sample points compared to background locations with constraints derived from the data [30]. The Maxent algorithm is deterministic and will converge to the probability distribution [28]. The model results in a nonnegative value assigned to each pixel of the study area grid, with values ranging from 0 to 1 to indicate the probability of occurrence for a given species/taxon of interest.

Maxent offers several advantages that are especially applicable to datasets like ours [30]. It relates presence locations to software generated pseudo-absence locations rather than to inferred absences collected in the field, an important factor when lacking actual absence data over a large spatial scale such as in this study. Further, Maxent is relatively insensitive to small spatial errors associated with location data and requires relatively few presence locations. It can employ both continuous and categorical response variables.

### Predictor Variables

We obtained data for environmental variables that uniformly covered our Greater Antilles study area and that we believed *a priori* would be potentially important predictors of Bicknell’s Thrush habitat. These environmental predictors included nine landform, land cover and climatic variables. We conducted all spatial data management and analysis with ESRI ArcGIS 10 software.

We used a global 30 arc-second (approximately 1 km^2^ resolution) digital elevation model from the NASA Shuttle Radar Topographic Mission (SRTM; http://srtm.csi.cgiar.org/). Slope and aspect were derived using surface analysis in ArcGIS Spatial Analyst. Aspect values were categorized as flat, north (316° to 45°), east (46° to 135°), south (136° to 225°), and west (226° to 315°).

Regional land cover data for the entire study area were lacking. We used landcover date from GlobCover version 2.2, which was the highest resolution (300 m) global land cover product ever produced and independently validated. This dataset was derived from an automatic and regionally-tuned classification of a time series of MERIS France composites from December 2004– June 2006. Its 22 land cover classes are defined with the United Nations Land Cover Classification System. Independent verification of classification accuracy was 73% globally [31], and forest classes performed best.

We used the high-resolution WorldClim dataset, version 1.4 (www.worldclim.org) [32]. These data correspond to the average climate conditions between ∼1950 and 2000, with most data from 1960–1990. Monthly total precipitation and monthly mean, minimum and maximum temperature were generated through interpolation of average monthly climate data from weather stations on a 30 arc-second resolution grid. Data uncertainty was estimated to be highest in mountainous areas (particularly for precipitation) and areas of low weather station density [32]. However, most of our study area appeared to have a relatively high density of weather stations.

We required several derivative climatic metrics that corresponded to the wintering period of Bicknell’s Thrush. We defined this period as November through March. Although timing and annual variability of the species’ wintering grounds arrival and departure are poorly known, our anecdotal observations indicate that birds arrive as early as mid to late October and depart in late April or early May. Because of this imprecision, we excluded these three months from the wintering period in our analyses. To generate winter mean temperature (tmean) grids, we took the arithmetic mean of the monthly maximum and minimum grids. Minimum (tmin) and maximum (tmax) mean winter temperature grids were calculated from monthly means. Winter mean precipitation (wprecip) was the sum of total mean monthly precipitation. We also used mean annual precipitation (precipyr) in our models.

### Model Selection

Following Yost [33], our objective was to build a model with adequate performance using the best subset of predictors. Employing the principle of parsimony, we defined the best model as that which contained the fewest predictor variables, with an average training gain not significantly different than the full model or the model with the highest training gain using the overlap in 95% confidence intervals as the criteria for significance [28], [33].

We used Maxent’s jackknife test of variable importance to evaluate the relative strengths of each predictor variable. The variable with the lowest decrease in the average training gain when omitted was removed and the remaining variables were used to build and run another model. This was repeated until we arrived at a two-variable model.

Model performance was evaluated by setting aside a subset of the presence records for training and using the remaining records to test the resulting model. Because performance can vary depending upon the particular set of data withheld from building the model for testing, we used 10 random partitions of the presence records to assess the average behavior of Maxent [28], [33]. For each partition, we randomly selected 75% of the 92 known Bicknell’s Thrush pixels, and we treated 10,000 random background pixels as negative instances for training data. The remaining 25% (n = 23) were used to test the model. The average training gain was calculated for each model from the 10 partitions.

Linear, quadratic, product, and hinge functions of the predictor variables were selected for inclusion in the models. Maximum number of iterations for the algorithm to approach convergence was set at 1000 with the threshold at 0.00001, and the regularization multiplier remained at the default value of one. All Bicknell’s Thrush presence localities were used to build the final model to create the estimated probability distribution map for use in a GIS.

Receiver operating characteristics (ROC) analysis were also used to evaluate how well the models compared to random prediction [30], [33]. The ROC plot shows how well the model correctly predicts presence (sensitivity) and absences (specificity). The significance of the ROC plot is quantified using the area under the curve (AUC), a ranked approach that determines the probability that a presence location will be ranked higher than a random background point.

We used the 10 percentile training presence logistic threshold to create a distribution map of potential Bicknell’s Thrush habitat. We used this map to estimate the amount of potential habitat within protected areas in each country, and we compared these to the extent of habitat outside protected areas and therefore presumably more vulnerable to future loss. The Nature Conservancy, which has produced the most recent information, provided protected areas boundaries for our study area. We used both designated and newly proposed (mostly Haiti) protected areas for our analysis as a best-case scenario.

## Results

We documented the presence of Bicknell’s Thrush at 179 sites representing 92 pixels on our study area grid. Of the 99 sites at which observers recorded vegetation type, 93% were in broadleaf forest, 4% in mixed pine-broadleaf forest, and 3% in dense pine forests. Most (82%) sites were categorized as wet, while 16% of sites were characterized as semi-humid and 2% as dry based on the presence of plant indicators (e.g., moss, tree ferns, lianas) and forest structure.

**Figure 1:**
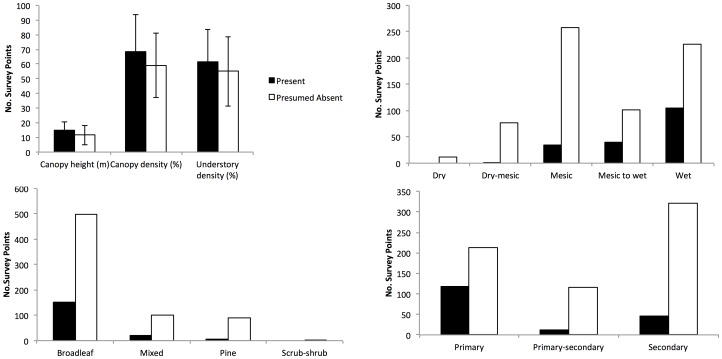
Ocular estimates of canopy height (m; mean±SD ), understory and canopy density (% cover; mean±SD), and the frequency of forest type, seral stage, and moisture regime at presence/presumed absence survey points for Bicknell’s Thrush in the Dominican Republic.

We detected thrushes at only 20.7% of survey points in the Dominican Republic where more detailed habitat metrics were recorded, highlighting the rarity of this species across the landscape. Points where Bicknell’s Thrush were present had higher canopy heights (*t* = −5.12, *p*<0.0001) and canopy densities (*t* = −4.48, *p*<0.0001), and a denser understory (*t* = −2.95, *p*<0.0001; Fig. 1) than points where thrushes were not detected. Most presence points were in broadleaf (85%) or mixed (12%) forests; mature seral stages and wetter sites were significantly more often occupied than younger and drier sites (Fig. 1).

**Figure 2:**
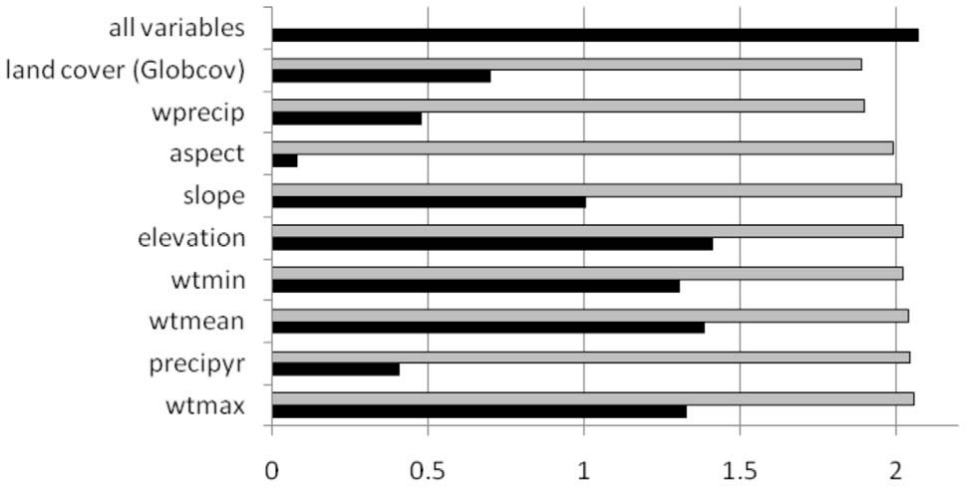
Training gain for each predictor variable alone (black) and the loss in training gain when the variable is removed from the full model (gray).

The regularized training gain for the full model with all presence records was 2.047. From the jackknife test of variable importance (Fig. 2), elevation was the most important predictor variable as measured by the gain produced by a one-variable model, closely followed by winter temperature variables. The two variables that decreased model gain the most when omitted from the full models were land cover and wprecip, suggesting that these two variables contained the most information not present in other predictor variables.

The average training gain generally declined as variables were removed (Table 1). Test AUC values were much better than random (>0.5 AUC) for all models (0.84–0.96 AUC), suggesting little model over-fitting. Using the overlap between 95% confidence intervals, both the average training gain and the average test AUC pointed to the same model as the most parsimonious.

**Table 1:** Maximum entropy general and reduced models using Globcover (2004–2006) land cover data to estimate Bicknell’s Thrush winter habitat in the Greater Antilles.

Model Variables	Training Gain	Test Gain	Test AUC
land cover, wprecip, aspect, slope, elev, wtmin, wtmean, precipyr, wtmax	2.069 (2.008–2.129)	2.060 (1.85–2.270)	0.942 (0.926–0.957)
land cover, wprecip, aspect, slope, elev, wtmin, wtmean, precipyr	2.041 (1.985–2.098)	2.204 (1.984–2.423)	0.955 (0.941–0.970)
land cover, wprecip, aspect, slope, elev, wtmin, wtmean	2.035 (1.983–2.087)	2.230 (2.025–2.436)	0.961 (0.952–0.970)
land cover,w precip, aspect, slope, elev, wtmin	2.031 (1.977–2.084)	2.140 (1.964–2.316)	0.952 (0.941–0.964)
**land cover, wprecip, aspect, slope, elev**	**1.963 (1.903–2.024)**	**1.955 (1.769–2.141)**	**0.942 (0.928–0.957)**
land cover, wprecip, aspect, slope	1.580 (1.502–1.659)	1.490 (1.232–1.748)	0.907 (0.891–0.924)
land cover, wprecip, aspect	1.044 (0.998–1.090)	0.913 (0.744–1.082)	0.862 (0.843–0.881)
land cover, wprecip	1.014 (0.962–1.065)	0.773 (0.593–0.953)	0.842 (0.819–0.865)

Values reported include training gain, test gain and test area under curve (AUC) averaged (95% confidence intervals) across 10 random partitions of presence data. Box indicates models that are not statistically different using the overlap between 95% confidence intervals. The best model based on parsimony is indicated in bold.

**Figure 3:**
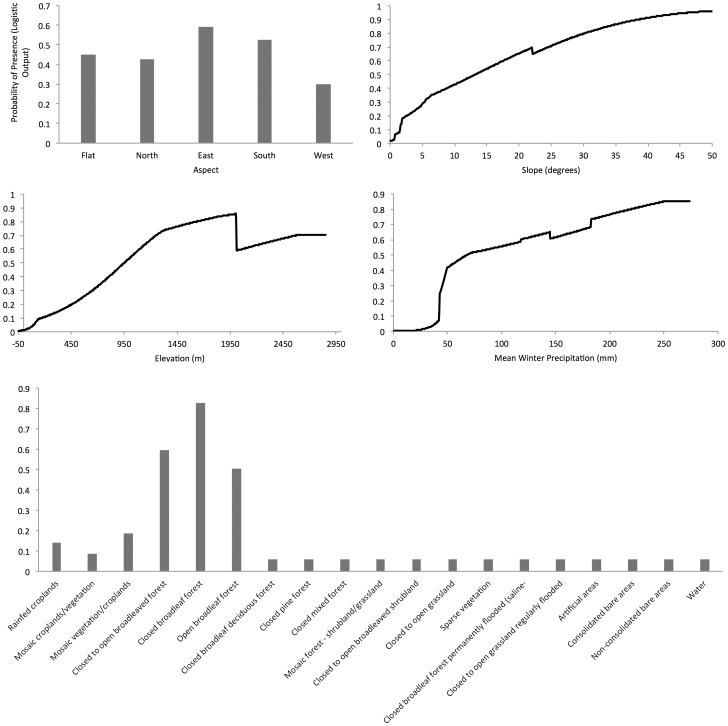
Response curves from a Maxent model created using only the corresponding variable for the model using GlobCov v2.2 (2004–2006) land cover data. These curves reflect the dependence of predicted suitability both on the selected variable and on dependencies induced by correlations between the selected variable and other variables.

A five-variable model with land cover, wprecip, aspect, slope, and elev was the most parsimonious and not significantly different than the four larger models (Table 1). This model had an AUC of 0.96. Elevation had the greatest contribution (59.2%) to the model followed by land cover (18.4%), wprecip (11.1%), slope (8%), and aspect (3.4%). Response curves indicated that increased probability of thrush presence was associated with higher elevations (increasing probability peaking at 2005 m), Globcover categorized as broadleaf forests (the highest being closed broadleaf forest), wprecip with higher probabilities generally above 67 mm, and steeper, non-west-facing slopes (Fig. 3).

**Figure 4:**
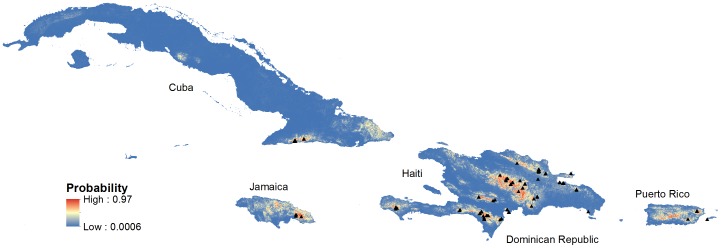
Maxent logistic estimates of probability of presence of Bicknell’s Thrush in the Greater Antilles. Black triangles indicate known locations Bicknell’s Thrush. Response variables included elevation, aspect (categorical), land cover (categorical), total winter precipitation, and winter mean minimum temperature.

We used the five-variable model constructed from the full set of 92 presence locations to create a distribution map of potential Bicknell’s Thrush habitat in the Greater Antilles (Fig. 4). Visual inspection showed strong correlation between known thrush locations and the continuous probability distribution. This was not surprising as the 10 percentile training presence logistic threshold was 0.25 and only six of the nine thrush locations below the 10 percentile had a logistic prediction of less than 0.20.

**Figure 5:**
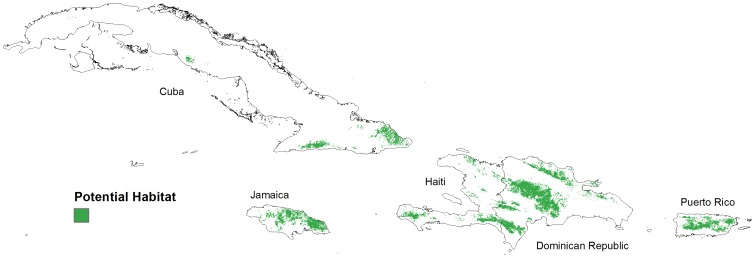
Potential winter habitat for Bicknell’s Thrush using the 10 percentile training presence logistic threshold (≥0.25) from the best-fitting Maxent model.

Potential Bicknell’s Thrush habitat was estimated to cover 33,170 km^2^, nearly 10% of the land in our study area (Fig. 5). The Dominican Republic contained just over half of all potential habitat (51%), followed by Cuba (15.1%), Jamaica (13.5%), Haiti (10.6%), and Puerto Rico (9.9%) (Table 2). Nearly one-third of the potential range was found to be in protected areas, with Cuban containing nearly 40% in protected areas to a low of under 10% in Puerto Rico. With a high proportion of the total habitat, 33% of the Dominican Republic thrush habitat is in some type of protected area.

**Table 2:** The amount of potential Bicknell’s Thrush habitat (km2) in protected and unprotected areas estimated from the most parsimonious Maxent model (land cover, wprecip, aspect, slope, and elev).

	% Total Habitat	Protected	Unprotected
Dominican Republic	51.0	5,587	11,314
Cuba	15.1	1,903	3,100
Jamaica	13.5	886	3,600
Haiti	10.6	745	2,764
Puerto Rico	9.9	324	2,947
Total		9,445	23,725

We used the 10 percentile training presence logistic threshold (0.248) to create a distribution map of potential Bicknell’s Thrush winter habitat. The Nature Conservancy provided up-to-date protected areas boundaries for our study area. We used both officially designated and newly proposed protected areas for our analysis as a best-case scenario.

## Discussion

Understanding distributional patterns of organisms is a fundamental question in conservation biology. Conservation planning and implementation require identifying pertinent habitats and locating geographic locations where land protection and management may improve viability of threatened species. This study provides the first detailed predictive map of Bicknell’s Thrush winter distribution and habitat suitability. Maximum entropy modeling of its geographic distribution effectively detailed the probability of Bicknell’s Thrush potential habitat at an approximately 1 km^2^ resolution across the Greater Antilles using nine environmental response variables that we believed *a priori* could best describe its habitat across this extensive landscape. The AUC score for most models was >0.90, which is considered to be very good [30].

Total winter precipitation, slope aspect and land cover, particularly broadleaf forests, emerged as important environmental variables. Moisture regime across the landscape was an important predictor, and the other top variables added information not contained in winter precipitation alone. However, moisture levels are likely indirectly correlated to other predictor variables. For example, slopes with western aspects tended to have the lowest probability of thrush presence. Major mountain ranges on each island force moisture-laden easterly trade winds to higher altitudes where most moist, submontane and lower montane wet and rain forests, including cloud forest formations, occur. Precipitation markedly decreases in the rain shadows of west-facing slopes in these mountainous areas [34], [35].

Probability for the elevation response curve peaked at 2,005 m and then sharply decreased to lower probabilities. This may in part reflect the existence of a discrete compositional ecotone between cloud forest and pine forest, as documented in the Dominican Republic’s Cordillera Central between 2,200 and 2,500 m elevation [35], [36]. Temperature, humidity and fire history, possibly influenced by the position of trade wind inversions, are thought to control the boundaries of this ecotone [35], which may vary elevationally among different mountain ranges within the Greater Antilles.

There is estimated to be 50,961 km^2^ of Bicknell’s Thrush breeding habitat in northeastern North America [6]. Our estimate of potential thrush winter habitat indicates a total land area of 33,170 km^2^. While methods do not allow for an exact comparison, potential habitat on the species’ winter range is approximately two-thirds of the extent of estimated U.S. and Canadian breeding habitat. The concentration of wintering Bicknell’s Thrushes into a smaller total area of suitable habitat may be partially accommodated by differences in winter and breeding spacing patterns and behavior. Wintering birds of all sex and age classes occupy exclusive territories that are significantly smaller than breeding home ranges, which are variable and often overlap extensively [2], [23]. In high- and mid-elevation broadleaf forests of the Dominican Republic, female thrushes territories averaged 1.07 ha±0.16 SE, while those of males covered 1.56 ha±0.17 [23]. In marked contrast, breeding birds in montane coniferous forests occupy much larger home ranges with males covering over 4 ha and females over 2 ha [2], However, these differences in spatial use and behavior might not be sufficient to enable the global population of Bicknell’s Thrush, estimated at 95,000–126,000 individuals [37], to occupy the smaller area of available habitat on its Greater Antillean winter range, especially given that the amount of available winter habitat is thought to have declined drastically due to relatively recent deforestation. Loss of suitable winter habitat may be at least partially responsible for observed population declines in the breeding grounds.

Model predictions typically project larger areal extents than the apparent realized distribution, because few species occupy all areas that satisfy their niche requirements [28]. Our extensive field experience throughout the Greater Antilles indicates that not all seemingly suitable broadleaf forest is occupied by Bicknell’s Thrush, and that thrush densities can vary from one forest patch or discrete forest type to another. It may also be a question of scale. On the breeding grounds both local and landscape scales were found to be important in determining changes in occupancy patterns [38]. An interaction between local and landscape parameters was detected for occupancy dynamics indicating that the relationship of the parameters to local-scale habitat conditions can change depending on the landscape context and vice versa [38]. It will be important to further validate and refine our models in the future, as well as to determine small scale response variables that influence Bicknell’s Thrush distribution and density.

The Bicknell’s Thrush presence data used in this study were compiled from disparate sources that included historic records back to 1975 and targeted surveys conducted between 1994–2009. It is difficult to adhere to standardized protocols that involve many contributors over such a long period of time, especially given the logistic challenges of accessing and working within potential Bicknell’s Thrush winter habitat. Our surveys may have been biased by unequal accessibility of field sites and by variations in sampling effort across space and time; we did not quantify these factors, which can affect predictive modeling [28]. Additionally, large errors within predictor variables can affect model accuracy [33].

The paucity of detailed historic data from the winter range of Bicknell’s Thrush obscures accurate evaluation of changes in distribution. We found that seven of 11 identifiable historic sites of the species’ occurrence in the Dominican Republic still supported thrushes during follow-up surveys conducted after 1995 [2]. However, several historic sites were severely degraded to the point of being unsuitable for thrush occupancy. Although historic deforestation rates can rarely be determined with accuracy, recent estimates in the Greater Antilles place overall loss of original forest cover at >90% in the Dominican Republic, >98% in Haiti, ∼ 75% in Jamaica, ∼ 80% in Cuba, and ∼50% in Puerto Rico [39]–[42]. High rates of winter habitat loss are believed to pose the most serious range-wide limiting factor for Bicknell’s Thrush [2], [37]. Given the species’ restricted and fragmented distribution at both ends of its migratory range, historic and ongoing forest loss throughout the Greater Antilles, and the potential for breeding and winter habitat perturbations from climate change [10], [43], we believe that Bicknell’s Thrush faces a tenuous future.

Less than 30% of the predicted winter habitat of Bicknell’s Thrush occurs within protected areas and many of these areas receive little actual on-the-ground protection or management. Thus, the majority of the species’ winter habitat occurs outside of direct governmental jurisdiction or in protected areas that are also threatened. We believe that efforts to protect and manage this species on the winter grounds will be most successful if the focus of conservation is aimed towards improving management of those areas already protected and identified as important Bicknell’s Thrush habitat, as well as expanding protected areas into regions identified as potential habitat hotspots. Given realities of limited budgets for conservation projects, we believe this model could be used by conservation practitioners to determine the greatest benefit to the species at the lowest cost.

Our results highlight the need for further efforts to conserve the winter habitats of this globally rare and vulnerable species. Actions recommended by the International Bicknell’s Thrush Conservation Group [37] on the wintering grounds of Bicknell’s Thrush include: (1) strengthening local protection of currently occupied habitat; (2) developing habitat management plans and restoration projects; (3) documenting overwinter survivorship and demography in relation to local habitat quality; and (4) extending surveys beyond Hispaniola to further clarify distribution and habitat use on Cuba, Jamaica and Puerto Rico [37]. This latter goal is crucial to prioritize and direct conservation planning. The montane forests preferred by Bicknell’s Thrush are considered to be among the most highly endangered forests in the Neotropics [36], [42]. Montane and cloud forests of the Greater Antilles support exceptionally high rates of endemism; 22 of Hispaniola’s recognized 31 endemic bird species are common residents of the mid- and high-elevation broadleaf forests occupied by Bicknell’s Thrush, and nine of these species are restricted to such forests [44]. Effective conservation of montane forests throughout the Greater Antilles will extend far beyond Bicknell’s Thrush to encompass a rich suite of biotic diversity.
